# Optimal Task Allocation Algorithm Based on Queueing Theory for Future Internet Application in Mobile Edge Computing Platform

**DOI:** 10.3390/s22134825

**Published:** 2022-06-25

**Authors:** Yukiko Katayama, Takuji Tachibana

**Affiliations:** Graduate School of Engineering, University of Fukui, 3-9-1 Bunkyo, Fukui 910-8507, Japan; yukiko-k@network.fuis.u-fukui.ac.jp

**Keywords:** mobile edge computing, future internet application, optimization problem, task allocation, heuristic algorithm, queueing theory

## Abstract

For 5G and future Internet, in this paper, we propose a task allocation method for future Internet application to reduce the total latency in a mobile edge computing (MEC) platform with three types of servers: a dedicated MEC server, a shared MEC server, and a cloud server. For this platform, we first calculate the delay between sending a task and receiving a response for the dedicated MEC server, shared MEC server, and cloud server by considering the processing time and transmission delay. Here, the transmission delay for the shared MEC server is derived using queueing theory. Then, we formulate an optimization problem for task allocation to minimize the total latency for all tasks. By solving this optimization problem, tasks can be allocated to the MEC servers and cloud server appropriately. In addition, we propose a heuristic algorithm to obtain the approximate optimal solution in a shorter time. This heuristic algorithm consists of four algorithms: a main algorithm and three additional algorithms. In this algorithm, tasks are divided into two groups, and task allocation is executed for each group. We compare the performance of our proposed heuristic algorithm with the solution obtained by three other methods and investigate the effectiveness of our algorithm. Numerical examples are used to demonstrate the effectiveness of our proposed heuristic algorithm. From some results, we observe that our proposed heuristic algorithm can perform task allocation in a short time and can effectively reduce the total latency in a short time. We conclude that our proposed heuristic algorithm is effective for task allocation in a MEC platform with multiple types of MEC servers.

## 1. Introduction

With the emergence of fifth generation (5G) mobile communication and Internet of Things, a variety of applications such as augmented reality, facial recognition, mobile game, smart city, and smart building, have been developed [1,2,3,4,5,6,7,8,9,10]. Many of these applications require high processing performance and low processing latency, and each task for these applications must be processed within an acceptable delay. However, it is difficult to process tasks for mobile applications within acceptable delays on mobile terminals [11,12,13]. This is because the processing capability of mobile terminals is low, and it takes a long time to process the tasks on these terminals.

By using task offloading, tasks for mobile applications can be processed on cloud servers, which are external servers with higher processing performance than mobile terminals [14]. The tasks can be processed on cloud servers in a short time [15]; however, the transmission delay is large due to the large distance between the mobile terminal and cloud servers [16]. Task offloading is a complex process and can be affected by a number of different factors [17], and it requires application partitioning, offloading decision making and distributed task execution [18].

Mobile edge computing (MEC) has attracted attention for processing tasks for applications that require low processing delay [19,20]. MEC was defined by the European Telecommunication Standards Institute [21], and it is also recently called Multi-Access Edge Computing. MEC is classified into one of the edge computing, which can support several kinds of characteristics including mobility support, location awareness, low latency, and heterogeneity [22,23,24]. In general, edge computing has more limited resources, limited computation and storage capabilities, and proximity to end devices than fog computing [25].

In a MEC platform in which MEC servers can be used, tasks can be processed on the MEC servers using task offloading, and the transmission delay for the task processing can be significantly reduced compared with cloud servers. However, the amount of available computing resources in a MEC server is limited, and the processing performance of a MEC server is lower than that of cloud servers. Thus, the number of tasks processed on a MEC server affects the performance of the MEC server. To process each task within an acceptable delay, some tasks should not be processed on the MEC server to avoid reducing the processing performance.

In some MEC platforms, MEC servers and cloud servers can be utilized for processing tasks [26]. MEC servers are classified into the following two groups based on the location and users: dedicated MEC servers and shared MEC servers. Dedicated MEC servers are utilized to process tasks that are sent from the closest access point, while shared MEC servers are utilized for tasks that are sent from any access point. Each task should be processed on an appropriate server among dedicated MEC servers, shared MEC servers, and cloud servers to satisfy the acceptable delay. Moreover, each task should be processed with low latency even if the acceptable delay is satisfied. Therefore, the total delay between sending a task and receiving a response for all tasks can be reduced by using MEC servers and cloud servers appropriately. However, the latency for each task is significantly affected by other task processes; therefore, it is difficult to perform task allocation for these servers. In addition, tasks transmitted from multiple access points are allocated to one of multiple MEC servers. Task allocation must be performed for tasks transmitted from multiple access points; however, it is difficult to consider the bottleneck node in an MEC platform. As far as the authors know, task allocation has not been studied in an MEC platform in which MEC servers and cloud servers are utilized from multiple access points and there is a bottleneck node.

In this paper, we propose a task allocation method for reducing the total latency in a MEC platform. In this platform, there are three types of servers: a dedicated MEC server, a shared MEC server, and a cloud server. For this platform, we first calculate the delay between sending a task and receiving a response for the dedicated MEC server, shared MEC server, and cloud server by considering the processing time and transmission delay. Here, the bottleneck node is modeled as an M/M/1 queueing model, and the transmission delay for the shared MEC server is derived using a queuing theory. Then, we formulate an optimization problem for task allocation to minimize the total latency for all tasks. By solving this optimization problem, tasks can be allocated to the MEC servers and cloud server appropriately. However, the calculation time is very large even if a meta-heuristic algorithm, such as the genetic algorithm [27], is used. Therefore, we also propose a heuristic algorithm to obtain the approximate optimal solution in a shorter time. This heuristic algorithm consists of four algorithms: a main algorithm and three additional algorithms. In this algorithm, tasks are divided into two groups, and task allocation is executed for each group. We compare the performance of our proposed heuristic algorithm with the solution obtained by the genetic algorithm and other methods and investigate the effectiveness of our algorithm.

Various studies have been conducted on task allocation methods for the MEC platform [20,26,28,29,30,31,32,33,34,35,36,37,38,39,40,41,42,43,44,45,46,47,48,49,50,51,52,53,54,55,56,57,58,59], which are described in Section 2. In comparison with these studies, we offer the following contributions and benefits:

- This paper considers task allocation for a MEC platform in which two types of MEC servers and a cloud server can be utilized.
- Three different equations are formulated to calculate the latency for each server.
- Our proposed heuristic algorithm can quickly derive the approximate optimal solution for the optimization problem in a situation in which three different servers are utilized.
- Our proposed heuristic algorithm can be implemented in a MEC platform and a mobile application, such as our developed application and system [60,61], because this algorithm is not complex for implementation.

Task allocation may fall into the local minimum when our proposed heuristic algorithm is used because task allocation processes are simple so that it can be implemented in a MEC platform. However, we will avoid falling into the local minimum by adding random search technique (ARSET) and heuristic random optimization (HRO) [62]. It should be noted that this paper is an extension of our previous work [63].

The remainder of this paper is organized as follows. Section 2 presents related work on task allocation in a MEC platform. Section 3 describes our system model, and Section 4 formulates an optimization problem to reduce the total latency in the MEC platform. Section 5 proposes a heuristic algorithm for solving the optimization problem, and Section 6 calculates computational complexity of the heuristic algorithm. Section 7 presents numerical examples, and Section 8 concludes the paper.

## 2. Related Work

In this section, we introduce related work on task allocation in a MEC platform. In [20], an offloading algorithm was proposed for multiple users to perform the computation offloading in a MEC environment. In this environment, multi-channel radio interference was utilized for offloading, and the algorithm used game theory for task offloading. In [26], the authors studied resource allocation for a multi-user MEC offloading system based on time-division multiple access and orthogonal frequency-division multiple access. In [28], the authors proposed a task allocation in a hybrid non-orthogonal multiple access (NOMA) MEC system to reduce the processing delay and save the energy consumption. The proposed method formulates an optimization problem and utilizes a matching algorithm to obtain a better solution. In [29], the authors proposed a cooperative task allocation method to minimize the power consumption of mobile terminals in an environment with a MEC server and cloud server. In this environment, task processing can be performed on the MEC server near the base station via wireless communication. This method can also use cloud servers via optical line terminals or the Internet.

In [30], the authors defined a mathematical model of a MEC environment in which traffic flows can be managed. The proposed permissive underestimation system, which selects the destination server with the lowest latency, provides an effective solution for a MEC platform. In addition, in [31], the authors discussed how a MEC server can be used to realize serverless edge computing. Following the European Telecommunications Standards Institute (ETSI) MEC standard, two alternative design approaches were proposed to handle rapid changes in mobility and load conditions. Using numerical examples, it was demonstrated that the proposed approaches were effective in accommodating system changes in response time.

In [32], the authors proposed an optimization framework for computation offloading and resource allocation for a MEC environment with multiple servers. This framework can be used to minimize the total computational overhead. The individual computation decisions, transmit power of the users, and computation resources were optimized. MEC servers were utilized in this environment; however, cloud servers were not. In addition, this paper adopted a suboptimal approach by splitting the original problem into a computation offloading decision problem and a joint resource allocation problem.

In [33], the authors investigated a two-tier offloading method for multiple MEC servers in heterogeneous networks. In this method, the total computation overhead was minimized by solving a formulated optimization problem that was a mixed-integer nonlinear program problem. The original problem was also divided into a resource allocation problem and a computation offloading problem. In [34], the authors focused on a MEC platform in which there were two types of MEC serves: a near server and far server. In this platform, delay-sensitive tasks were allocated to the near server while computationally intensive tasks were allocated to the far server. However, this task allocation did consider the utilization of cloud servers. In [35], a resource management technique based on game theory was proposed in a MEC platform and small-scale data centers. This technique can minimize energy consumption and costs while ensuring applications’ performance using a semi-co-operative game.

In [36], the authors proposed a heuristic offloading algorithm to maximize the reliability performance of computation offloading. The method can be used in an Internet of Vehicle environment in which fixed edge computing node and MEC nodes are used, but cloud servers are not used. For a similar Internet of Vehicle environment, in [37], the authors modeled the data redundancy and proposed the collaborative task computing scheme. The proposed scheme can reduce the redundant data and utilize the idle resources in nearby MEC servers. In [38], the authors proposed an optimization framework of offloading from a single mobile device to multiple edge devices. This framework is based on a semi-definite relaxation (SDR), and tasks are allocated considering central process unit (CPU) to improve energy consumption and processing latency. In [39], for the industrial Internet of Things, the authors proposed a MEC-enabled architecture considering the task’s priority constraints. This architecture can minimize the response time using a task allocation strategy using a Bayesian network based evolutionary algorithm. In [40], for the latency and reliability sensitive computing tasks processed in swarm of drones, the authors proposed a task allocation based on an optimization problem. In the swarm of drones, nearby drones are used as MEC server for processing the tasks. This algorithm can minimize the energy consumption of the swarm of drones when the latency and reliability requirements are satisfied.

For cloud computing environments without MEC servers, in [41,42], the authors proposed resource management methods for cloud computing environments and cloud data centers. These methods can manage resources to improve energy consumption, service performance, and costs. In [43], the authors studied the combination of two virtualization technologies: virtual machine and containers. The authors presented the advantages of running containers on virtual machines.

For a environment where MEC servers and cloud servers are available, [44,45] proposed an algorithm that allocates tasks to a MEC server or cloud servers to minimize the total latency. Optimization problems were formulated for latency reduction and were solved using a genetic algorithm. In both problems, there was only one MEC server, and heuristic algorithms were not proposed. In [46] a task allocation to increase user satisfaction was proposed. The minimization of power consumption was also considered [47,48,49,50,51].

MEC is significantly expected to be utilized by future Internet applications. Therefore, various uses of MEC have been proposed [52,53,54,55]. Especially, machine learning and artificial intelligence are effective in a MEC platform [56,57,58,59]. For utilizing machine learning and artificial intelligence, a large number of data sets obtained from the real environment and a long training time to determine an appropriate task allocation.

## 3. System Model

### 3.1. MEC Platform

In this section, we explain our system model where our proposed method is applied. This system model is designed by considering [61] because our proposed method is used in real environments.

**Figure 1:**
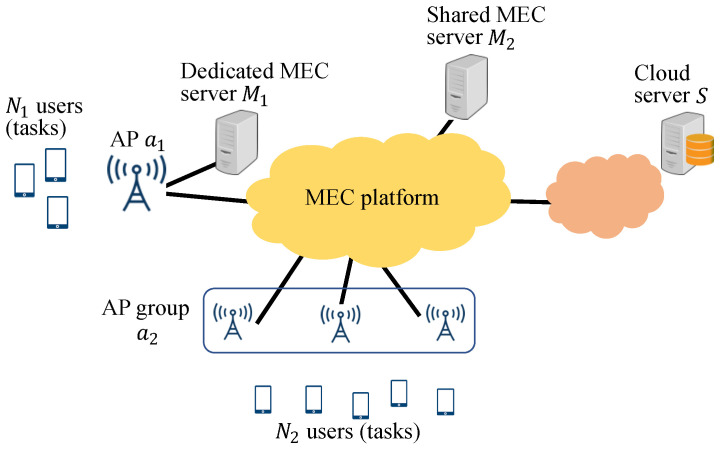
System model consisting of a MEC platform with three types of servers.

Figure 1 presents our system model, which consists of a MEC platform with three types of servers: a dedicated MEC server, shared MEC server, and cloud server. The dedicated MEC server $M_{1}$ is utilized to process tasks that are sent from the closest access point, while the shared MEC server $M_{2}$ is utilized for tasks that are sent from any access point. The cloud server *S* can also be utilized for tasks that are sent from any access point.

In this model, *N* tasks of *N* users can be processed on one of the three servers in the MEC platform. In the following, we focus on task allocation for users that connect to the MEC platform via access point $a_{1}$. The number of these users is $N_{1}$, and the $N_{1}$ users can use the dedicated MEC server $M_{1}$, shared MEC server $M_{2}$, and cloud server *S*. In addition, $N_{2}$ users ($N_{1}+N_{2}=N$) can use $M_{2}$ and *S* via access point $a_{2}$. That is, $M_{2}$ and *S* can process tasks for all *N* users, whereas $M_{1}$ can process tasks for $N_{1}$ users.

**Figure 2:**
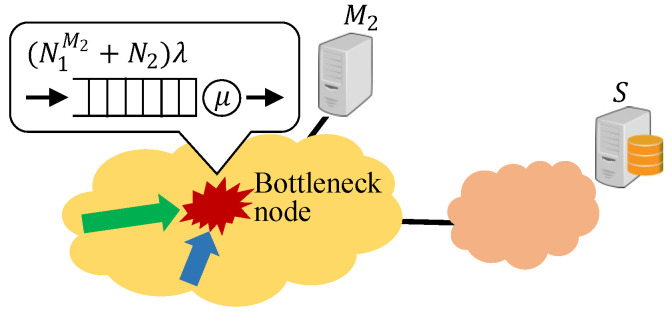
M/M/1 queueing model for bottleneck node in a MEC platform.

Here, let $D_{1}$ [Gigacycle/s] be the processing efficiency of $M_{1}$, and let $D_{2}$ [Gigacycle/s] be the processing efficiency of $M_{2}$. The transmission delay between $N_{1}$ users and $M_{1}$ is zero; however, the transmission delay between $N_{1}$ users and $M_{2}$ depends on the bottleneck node (see Figure 2). In this subsection, we model the bottleneck node between all access points and $M_{2}$ as an M/M/1 queueing model, and the transmission delay *l* [s] is given by

(1) $$l=\frac{1}{\mu -(N_{1}^{M_{2}}+N_{2})\lambda }.$$

In (1), $N_{1}^{M2}$ denotes the number of tasks that are not allocated to $M_{1}$, and $\left(N_{1}^{M2}+N_{2}\right)$ denotes the number of tasks that pass through the bottleneck node. In addition, λ is the arrival rate of tasks at the bottleneck node, and $\frac{1}{\mu }$ is the average processing time of each task at the bottleneck node. It should be noted that $(N_{1}^{M2}+N_{2})\lambda /\mu <1$ should be satisfied to obtain steady-state probabilities.

The processing efficiency of *S* is much higher than that of both $M_{1}$ and $M_{2}$; thus, the processing time on *S* is assumed to be 0 [s]. The transmission delay between $N_{1}$ users and *S* is large because the task transmission is via the Internet, and this transmission delay is assumed to be a large constant time, which is denoted as τ [s].

Here, let the *i*th task that is transmitted via access point $a_{1}$ be denoted as $f_{i}$ ($i=1,\cdots ,N_{1}$). For task $f_{i}$, the acceptable latency is set to $t_{i}^{max}$ [s] as the task allocation constraint. Each user must receive a response for their own task within the acceptable delay after sending the task to a server.

### 3.2. Calculation of Latency for Three Types of Servers

**Figure 3:**
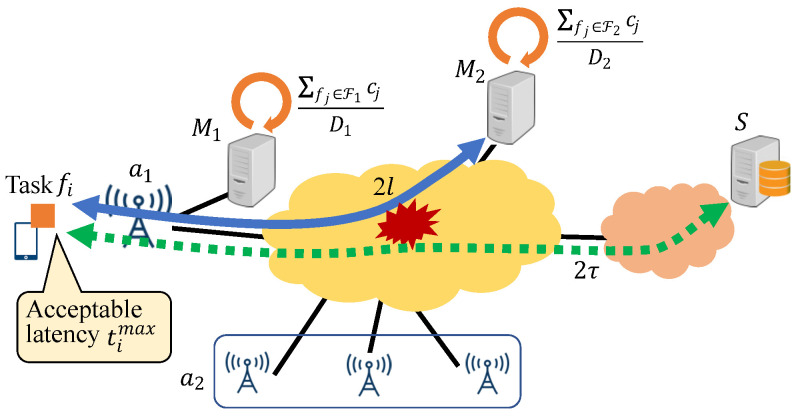
Latency for processing task $f_{i}$.

In this subsection, we calculate the latency for processing a task for three types of servers. For task $f_{i}$ ($i=1,\cdots ,N_{1}$), let $T_{1}^{i}$, $T_{2}^{i}$, and $T_{S}^{i}$ be the latency for processing $f_{i}$ on the dedicated MEC server $M_{1}$, shared MEC server $M_{2}$, and cloud server *S*, respectively. Figure 3 presents the latency for $f_{i}$ in the three cases.

Now, let $c_{i}$ [Gigacycle] denote the number of central processing unit (CPU) cycles that are required for processing $f_{i}$. When $f_{i}$ is processed on $M_{1}$, $T_{1}^{i}$ is equal to the processing time on $M_{1}$ and does not include the transmission delay. This is because the transmission delay is zero for $M_{1}$. Here, the processing time depends on the total number of CPU cycles for the tasks processed on $M_{1}$. When the set of tasks processed on $M_{1}$ is $F_{1}$, $T_{1}^{i}$ is given by

(2) $$T_{1}^{i}=\frac{\sum_{f_{j}\in F_{1}}c_{j}}{D_{1}}.$$

Next, we consider the processing of $f_{i}$ on $M_{2}$. The latency $T_{2}^{i}$ is derived from the processing time on $M_{2}$ and the round-trip transmission delay 2l, where *l* is derived in Section 3.1. Here, the processing time also depends on the total number of CPU cycles for the tasks processed on $M_{2}$. When the set of tasks processed on $M_{2}$ is $F_{2}$, the latency $T_{2}^{i}$ is given by

(3) $$T_{2}^{i}=\frac{\sum_{f_{j}\in F_{2}}c_{j}}{D_{2}}+2l.$$

It should be noted that for simplicity, the processing time, which is the first term in (3), does not consider the processing of tasks forwarded from $a_{2}$. This is because we do not focus on the allocation of tasks from $a_{2}$; however, this does not affect the allocation of tasks from $a_{1}$ because we assume that $D_{2}$ is the efficiency of processing only tasks from $a_{1}$.

Finally, when $f_{i}$ is processed on *S*, the latency $T_{S}^{i}$ is equal to the round-trip transmission delay 2τ. This is because the processing time of $f_{i}$ on *S* is zero due to its high processing efficiency regardless of the number of tasks that are allocated to *S*. Therefore, $T_{S}^{i}$ is given by

(4) $$T_{S}^{i}=2\tau .$$

## 4. Optimization Problem Formulation for Total Latency Reduction

In this section, we formulate an optimization problem for allocating tasks to three servers to minimize the total latency for the system model described in Section 3. For this optimization problem, we define the following variables for task $f_{i}$:

$$\chi_{i}=\left\{\begin{array}{cc} 1, & f_{i}\mathrm{is}\mathrm{allocated}\mathrm{to}M_{1},\\ 0, & \mathrm{otherwise}. \end{array}\right.$$

$$\psi_{i}=\left\{\begin{array}{cc} 1, & f_{i}\mathrm{is}\mathrm{allocated}\mathrm{to}M_{2},\\ 0, & \mathrm{otherwise}. \end{array}\right.$$

$$\omega_{i}=\left\{\begin{array}{cc} 1, & f_{i}\mathrm{is}\mathrm{allocated}\mathrm{to}S,\\ 0, & \mathrm{otherwise}. \end{array}\right.$$

The above variables indicate the server where $f_{i}$ is processed. For example, $\chi_{i}=1$ indicates that $f_{i}$ is allocated to $M_{1}$.

When the acceptable latency for $f_{i}$ is $t_{i}^{max}$, we formulate the following optimization problem for minimizing the total latency for all $N_{1}$ tasks:

(5) $$\min_{\chi ,\psi ,\omega }\;\;\;\sum_{i=1}^{N}\left\{T_{1}^{i}\chi_{i}+T_{2}^{i}\psi_{i}+T_{S}^{i}\omega_{i}\right\},$$

subject to:

(6) $$T_{1}^{i}\chi_{i}+T_{2}^{i}\psi_{i}+T_{S}^{i}\omega_{i}\leq t_{i}^{max},\;\forall i,$$

(7) $$\chi_{i}+\psi_{i}+\omega_{i}=1,\;\forall i.$$

In this optimization problem, the objective function (5) signifies that tasks are allocated to servers to minimize the total latency. The constraint condition (6) indicates that the latency for each task must be equal to or lower than $t_{i}^{max}$. Moreover, (7) signifies that each task is allocated to only one of three servers. This optimization problem can be solved simply using meta heuristic algorithms, such as the genetic algorithm.

## 5. Proposed Heuristic Algorithm

In this section, we propose a heuristic algorithm for solving the formulated optimization problem. Our proposed heuristic algorithm consists of four algorithms that are denoted as Algorithms 1–4. Algorithm 1 is the main algorithm, while the remaining algorithms are used as a function in the main algorithm.

**Figure 4:**
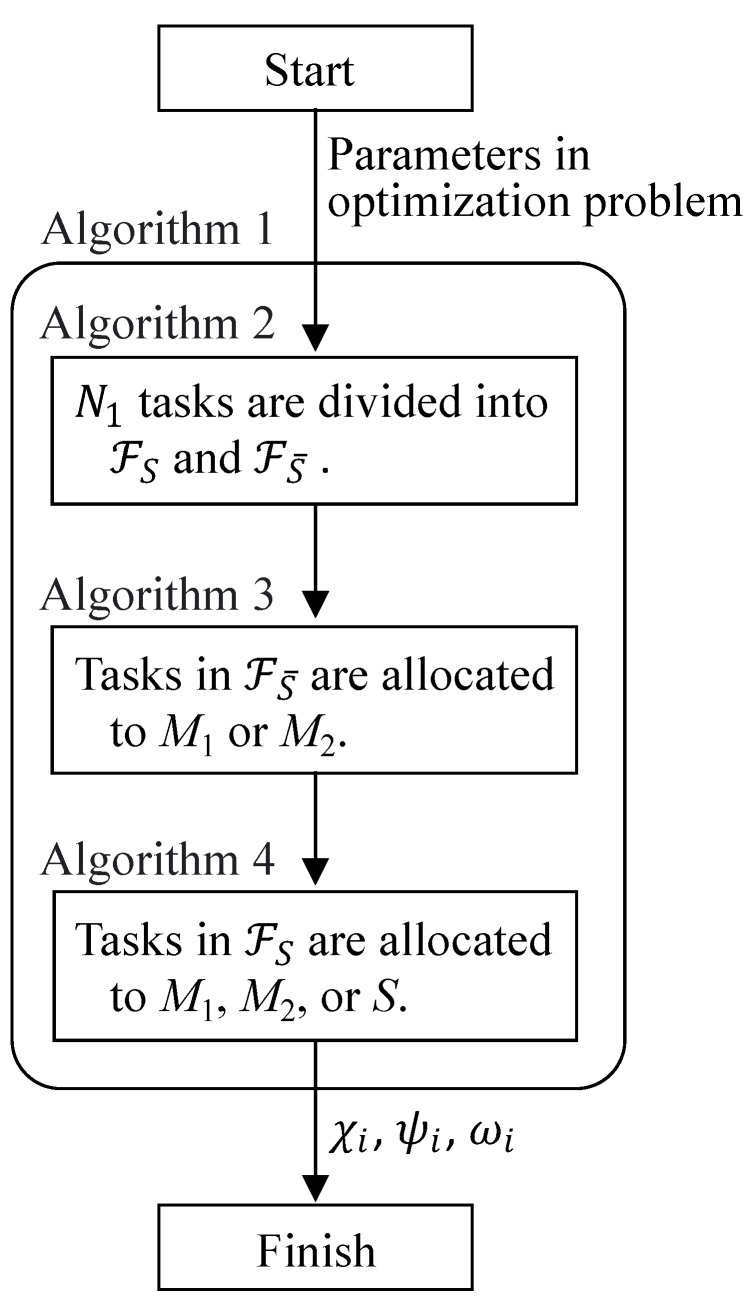
Overview of our proposed heuristic algorithm.

Figure 4 presents an overview of our proposed heuristic algorithm. In our algorithm, the allocation of a task whose acceptable latency is low is preferentially performed to satisfy the acceptable latency of all tasks. In Algorithm 1, first, all tasks are divided into two sets in line 1. This process is performed based on the acceptable latency in Algorithm 2.
**Algorithm 1** Main algorithm.**Input:**
All parameters for our optimization problem    **Output:**
$\chi_{i}$, $\psi_{i}$, $\omega_{i}$     1: Task division ($\left(t_{i}^{max},\tau ,f_{i}\right)$, $N_{1}$)      /*Algorithm 2*/
     2: MEC allocation $\left(t_{i}^{max},f_{i},c_{i}\right)$      /*Algorithm 3*/      3: MEC cloud allocation $\left(t_{i}^{max},f_{i},c_{i}\right)$   /*Algorithm 4*/

In Algorithm 2, tasks are divided into two sets, $F_{S}$ and $F_{\bar{S}}$. $F_{S}$ includes tasks that can be processed on *S*, while $F_{\bar{S}}$ includes tasks that are never processed on *S*. If the acceptable latency $t_{i}^{max}$ of task $f_{i}$ is smaller than 2τ, $f_{i}$ is never processed on *S* and is included in $F_{\bar{S}}$ in lines 3 and 4. Otherwise, $f_{i}$ is included in $F_{S}$ in lines 5 and 6.

Then, each task in $F_{\bar{S}}$ is allocated to $M_{1}$ or $M_{2}$ in line 2 of Algorithm 1, and this allocation is performed in Algorithm 3. In Algorithm 3, let $L_{i}^{1}$ and $L_{i}^{2}$ be the latency when $f_{i}$ is assumed to be processed on $M_{1}$ and $M_{2}$, respectively. Furthermore, $V_{min}^{1}$ and $V_{min}^{2}$ are the minimum values of the acceptable latency $t_{i}^{max}$ of a task allocated to $M_{1}$ and $M_{2}$, respectively. As explained in the previous paragraph, a task in $F_{\bar{S}}$ must be allocated to $M_{1}$ or $M_{2}$ because $t_{i}^{max}$ is smaller than 2τ. In addition, a task whose acceptable latency is low should be allocated to $M_{1}$ because the transmission delay for $M_{1}$ is zero. Therefore, the allocation of tasks in $F_{\bar{S}}$ is decided in ascending order of $t_{i}^{max}$, and $f_{i}$ is sorted in ascending order of $t_{i}^{max}$ in line 1. It should be noted that $t_{1}^{max}$ is the minimum value while $t_{|F_{\bar{S}}|}^{max}$ is the maximum value after line 1. In this task allocation, $L_{i}^{1}$ and $L_{i}^{2}$ are compared with $V_{min}^{1}$ and $V_{min}^{2}$ in lines 6, 18, and 23. When all tasks satisfy the acceptable latency even if $f_{i}$ is allocated to $M_{1}$ and $M_{2}$ in line 6, $f_{i}$ is allocated to a server to reduce the latency by comparing $L_{i}^{1}$ with $L_{i}^{2}$ in lines 7 or 12 ($\chi_{i}\leftarrow 1$ or $\psi_{i}\leftarrow 1$). After $f_{i}$ is allocated to a server, $V_{min}^{1}$ or $V_{min}^{2}$ may be updated in line 10 or 15. When all tasks satisfy the acceptable latency if $f_{i}$ is allocated to $M_{1}$ but the acceptable latency is not satisfied for $M_{2}$ in line 18, $f_{i}$ is allocated to $M_{1}$ ($\chi_{i}\leftarrow 1$). In addition, when all tasks satisfy the acceptable latency if $f_{i}$ is allocated to $M_{2}$, but the acceptable latency is not satisfied for $M_{1}$ in line 23, $f_{i}$ is allocated to $M_{2}$ ($\psi_{i}\leftarrow 1$). In both cases, $V_{min}^{1}$ or $V_{min}^{2}$ may be updated in line 21 or 26.
**Algorithm 2** Task division function.**Input:**$t_{i}^{max},\tau ,f_{i},N_{1}$   **Output:**$F_{S},F_{\bar{S}}$          *Initialization*:     1: i←0          *LOOP Process*:     2: **while**$i<N_{1}$**do**     3:       **if** $t_{i}^{max}<2\tau$ **then**     4:           $F_{\bar{S}}\leftarrow f_{i}$     5:       **else**     6:           $F_{S}\leftarrow f_{i}$     7:       **end if**     8:       i←i+1     9: **end while**

**Algorithm 3** MEC allocation function.**Input:**$t_{i}^{max},f_{i},c_{i}$ **Output:**$\chi_{i}$, $\psi_{i}$, $\omega_{i}$ for $f_{i}\in F_{\bar{S}}$       *Initialization*:  1: $f_{i}$ in $F_{\bar{S}}$ is sorted in ascending order of $t_{i}^{max}$  2: $V_{min}^{1}\leftarrow \infty$  3: $V_{min}^{2}\leftarrow \infty$  4: i←1      *LOOP Process*:  5: **while**
$i<|F_{\bar{S}}|$
**do**  6:       **if** $L_{i}^{1}\leq V_{min}^{1}$ and $L_{i}^{2}\leq V_{min}^{2}$ **then**  7:             **if** $L_{i}^{1}\leq L_{i}^{2}$ **then**  8:                  $\chi_{i}\leftarrow 1$  9:                  **if** $V_{min}^{1}>t_{i}^{max}$ **then** 10:                        $V_{min}^{1}\leftarrow t_{i}^{max}$ 11:                  **end if** 12:             **else** 13:                  $\psi_{i}\leftarrow 1$ 14:                  **if** $V_{min}^{2}>t_{i}^{max}$ **then** 15:                        $V_{min}^{2}\leftarrow t_{i}^{max}$ 16:                  **end if** 17:             **end if** 18:       **else if** $L_{i}^{1}\leq V_{min}^{1}$ and $L_{i}^{2}>V_{min}^{2}$ **then** 19:             $\chi_{i}\leftarrow 1$ 20:             **if** $V_{min}^{1}>t_{i}^{max}$ **then** 21:                  $V_{min}^{1}\leftarrow t_{i}^{max}$ 22:             **end if** 23:       **else** 24:             $\psi_{i}\leftarrow 1$ 25:             **if** $V_{min}^{2}>t_{i}^{max}$ **then** 26:                  $V_{min}^{2}\leftarrow t_{i}^{max}$ 27:              **end if** 28:       **end if** 29:  **end while**

In Algorithm 4, tasks are allocated to $M_{1}$, $M_{2}$, or *S* because $f_{i}$ can be allocated to the cloud server. Here, this task allocation can easily satisfy the acceptable latency for a task by allocating the task to *S*. This is because the processing time for $M_{1}$ and $M_{2}$ does not change when the task is allocated to *S*. Therefore, in this algorithm, $f_{i}$ in $F_{S}$ is sorted in descending order of $c_{i}$ to reduce the total latency in line 1. It should be noted that $c_{1}$ is the maximum value and $c_{|F_{S}|}$ is the minimum value after line 1. Here, let $K_{i}^{1}$, $K_{i}^{2}$, and $K_{i}^{S}$ be the total latency for $M_{1}$, $M_{2}$, and *S* in the case in which $f_{i}$ is assumed to be processed on $M_{1}$, $M_{2}$, and *S*, respectively. In this task allocation, $L_{i}^{1}$ and $L_{i}^{2}$ are compared with $V_{min}^{1}$ and $V_{min}^{2}$ in lines 6, 20, 29, and 38. When all tasks satisfy the acceptable latency, even if $f_{i}$ is allocated to $M_{1}$ and $M_{2}$ in line 6, $f_{i}$ is allocated to a server so that the total latency becomes the smallest in lines 7, 12, or 17 ($\chi_{i}\leftarrow 1$, $\psi_{i}\leftarrow 1$, or $\omega_{i}\leftarrow 1$). After $f_{i}$ is allocated to $M_{1}$ or $M_{2}$, $V_{min}^{1}$ or $V_{min}^{2}$ may be updated in line 10 or 15. When all tasks satisfy the acceptable latency if $f_{i}$ is allocated to $M_{1}$, but the acceptable latency is not satisfied for $M_{2}$ in line 20, $f_{i}$ is allocated to $M_{1}$ or *S*. In line 21 or 26, $f_{i}$ is allocated to a server so that the total latency becomes the smallest ($\chi_{i}\leftarrow 1$ or $\omega_{i}\leftarrow 1$). In addition, when all tasks satisfy the acceptable latency if $f_{i}$ is allocated to $M_{2}$, but the acceptable latency is not satisfied for $M_{1}$ in line 29, $f_{i}$ is allocated to $M_{2}$ or *S*. In line 30 or 35, $f_{i}$ is allocated to a server so that the total latency becomes the smallest ($\psi_{i}\leftarrow 1$ or $\omega_{i}\leftarrow 1$). When no task can satisfy the acceptable latency if $f_{i}$ is allocated to $M_{1}$ and $M_{2}$, $f_{i}$ is allocated to *S* ($\omega_{i}\leftarrow 1$).
**Algorithm 4** MEC cloud allocation function.**Input:**$t_{i}^{max},f_{i},c_{i}$   **Output:**$\chi_{i}$, $\psi_{i}$, $\omega_{i}$ for $f_{i}\in F_{S}$          *Initialization*:     1: $f_{i}$ in $F_{S}$ is sorted in decreasing order of $c_{i}$     2: $V_{min}^{1}\leftarrow \infty$     3: $V_{min}^{2}\leftarrow \infty$     4: i←1          *LOOP Process*:     5: **while**
$i<|F_{S}|$
**do**     6:       **if** $L_{i}^{1}\leq V_{min}^{1}$ and $L_{i}^{2}\leq V_{min}^{2}$ **then**     7:             **if** $K_{i}^{1}<K_{i}^{2}$ and $K_{i}^{1}<K_{i}^{S}$ **then**     8:                  $\chi_{i}\leftarrow 1$     9:                  **if** $V_{min}^{1}>t_{i}^{max}$ **then**   10:                        $V_{min}^{1}\leftarrow t_{i}^{max}$   11:                  **end if**   12:             **else if** $K_{i}^{2}<K_{i}^{1}$ and $K_{i}^{2}<K_{i}^{S}$ **then**   13:                  $\psi_{i}\leftarrow 1$   14:                  **if** $V_{min}^{2}>t_{i}^{max}$ **then**   15:                        $V_{min}^{2}\leftarrow t_{i}^{max}$   16:                  **end if**   17:             **else**   18:                  $\omega_{i}\leftarrow 1$   19:             **end if**   20:       **else if** $L_{i}^{1}\leq V_{min}^{1}$ and $L_{i}^{2}>V_{min}^{2}$ **then**   21:             **if** $K_{i}^{1}<K_{i}^{S}$ **then**   22:                  $\chi_{i}\leftarrow 1$   23:                  **if** $V_{min}^{1}>t_{i}^{max}$ **then**   24:                        $V_{min}^{1}\leftarrow t_{i}^{max}$   25:                  **end if**   26:             **else**   27:                  $\omega_{i}\leftarrow 1$   28:             **end if**   29:       **else if** $L_{i}^{1}>V_{min}^{1}$ and $L_{i}^{2}\leq V_{min}^{2}$ **then**   30:             **if** $K_{i}^{2}<K_{i}^{S}$ **then**   31:                  $\psi_{i}\leftarrow 1$   32:                  **if** $V_{min}^{2}>t_{i}^{max}$ **then**   33:                        $V_{min}^{2}\leftarrow t_{i}^{max}$   34:                  **end if**   35:             **else**   36:                  $\omega_{i}\leftarrow 1$   37:             **end if**   38:       **else**   39:             $\omega_{i}\leftarrow 1$   40:       **end if**   41:       i←i+1   42: **end while**

## 6. Computational Complexity

In order to investigate the scalability of our proposed algorithm, we derive computational complexity of our proposed heuristic algorithm. First, there is no loop process in Algorithm 1, which is the main algorithm; therefore, the computational complexity of this algorithm can be derived from Algorithm 2, Algorithm 3, or Algorithm 4.

In Algorithm 2, there is a loop process from line 2 to line 9, and the order of this loop process is O(N), in which *N* is the number of tasks. In Algorithms 3 and 4, there is also a loop process from line 5 to line 29 and from line 5 to line 42, respectively. From line 5 of Algorithm 3, the order of this loop process is O(N) because $|F_{\bar{S}}|$ is equal to or smaller than *N*. Moreover, from line 5 of Algorithm 4, the order of this loop process is also O(N) because $|F_{S}|$ is equal to or smaller than *N*.

As a result, the computational complexity of our proposed algorithm is O(1)×(O(N)+O(N)+O(N))=O(N). This signifies that the computational complexity of this algorithm does not depend on parameters of a MEC platform and is affected by only the number of tasks. Therefore, our proposed algorithm is scalable to a large-scale MEC platform.

## 7. Numerical Examples

In this section, we evaluate the performance of our proposed heuristic algorithm described in Section 5 through comparison with other methods such as near-optimal task allocation with the genetic algorithm.

In the MEC platform for the performance evaluation, the number of tasks $N_{1}$ is 10, 20, 30, 40, or 50, and the number of tasks $N_{2}$ is equal to 20. The processing efficiency for $M_{1}$ is $D_{1}=30$, and the processing efficiency for $M_{2}$ is $D_{2}=300$. In addition, the transmission delay of tasks for *S* is τ=0.1, 0.2, 0.3, 0.4, or 0.5. For task $f_{i}$, $c_{i}$ is determined according to a uniform distribution of [0.1,1.0], and $t_{i}^{max}$ is determined according to a uniform distribution of [1.0,4.0]. For the bottleneck node, we assume that λ is equal to 1.25, 1.5, 1.75, 2.0, or 2.25, and μ is equal to 100. Table 1 presents a list of parameter settings in the simulation. These parameter settings were decided according to our MEC platform and application [62].

For this MEC platform, we evaluate the performance of the proposed heuristic algorithm, denoted as Proposed, and the performance of near-optimal task allocation, denoted as GA. In near-optimal task allocation, the number of chromosomes in each generation is 1000 and the mutation probability is 0.005. GA algorithm stops if there is no improvement in the best objective value for 1000 generations. It should be noted that we determined that the result of GA is the same as the optimal value obtained by the CPLEX optimizer [64] when the number of tasks is small. Therefore, the result of GA is used as the optimal one, and the performance of our proposed heuristic algorithm is investigated by comparing with the result of GA.

We also evaluate another heuristic algorithm where $K_{i}^{1}$, $K_{i}^{2}$, and $K_{i}^{S}$ are replaced by $L_{i}^{1}$, $L_{i}^{2}$, and 2τ in Algorithm 4. This signifies that the latency for $f_{i}$ is considered, but the total latency is not considered in Algorithm 4. The performance evaluation of this method is useful to investigate the validity of our proposed heuristic algorithm where the total latency can be considered, and the result is denoted as Comp. Finally, as one of the simplest methods, we evaluate the performance of a random method, this is denoted as Random, in which tasks are allocated to the three servers at random. We evaluate the performance of this method by deriving the average value from 10 simulations; the result is denoted as Random. By comparing Proposed with Random, the processing complexity of Proposed can be investigated.

In the following performance evaluation, there are four performance metrics:

- Total latency: The solution of (5).
- Minimum latency: $\min_{i}\left\{T_{1}^{i}\xi_{i}+T_{2}^{i}\psi_{i}+T_{S}^{i}\omega_{i}\right\}$.
- Maximum latency: $\max_{i}\left\{T_{1}^{i}\xi_{i}+T_{2}^{i}\psi_{i}+T_{S}^{i}\omega_{i}\right\}$.
- Calculation time to perform task allocation by solving the optimization problem.

These metrics are derived by solving the optimization problem (5) using the four methods.

### 7.1. Impact of Number of Tasks

**Figure 5:**
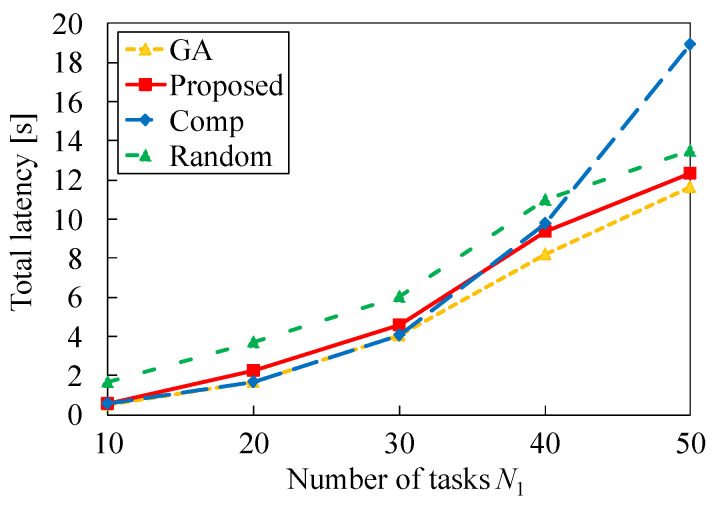
Total latency versus number of tasks in the case of τ=0.2 and λ=2.0.

First, we investigate the impact of the number of tasks *N* on the performance of each method when the transmission delay τ via the Internet is 0.2 and the arrival rate λ is 2.0. Figure 5 presents the total latency versus the number of tasks *N*. This figure indicates that the total latency increases as the number of tasks increases for all methods. This is because the total number of CPU cycles required for processing tasks increases. Among the four methods, the total latency of GA is the lowest, as expected. Furthermore, the latency of Random is much higher than that of GA, which demonstrates that tasks should not be allocated to servers at random. For our proposed method (Proposed), the obtained latency is close to the near-optimal result of GA. This is because our proposed method is constructed to obtain appropriate solution for the optimization problem (5)–(7). Moreover, the latency of Comp is almost the same as that of GA when the number of tasks is small; however, the latency increases as the number of tasks increases. When the number of tasks is 50, the total latency of Comp is much higher than that of Random. Therefore, Figure 5 demonstrates that our proposed heuristic algorithm can effectively reduce the total latency compared to Random and Comp.

**Figure 6:**
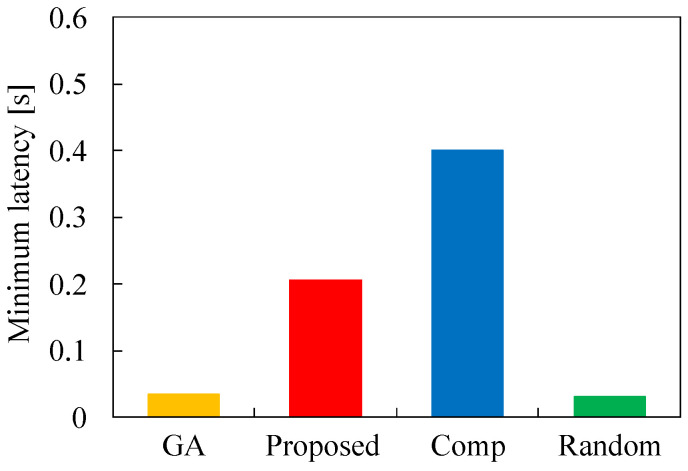
Minimum latency for each method in the case of $N_{1}=50$, τ=0.2, and λ=2.0.

**Figure 7:**
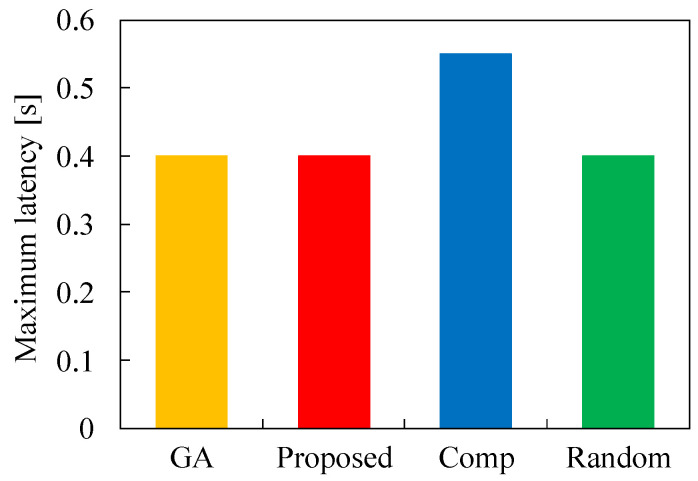
Maximum latency for each method in the case of $N_{1}=50$, τ=0.2, and λ=2.0.

Next, we evaluate the minimum latency and maximum latency for each task allocation in Figure 6 and Figure 7, respectively. In these figures, the number of tasks *N* is 50, τ is 0.2, and λ is 2.0. Figure 6 demonstrates that the minimum latency of Proposed is larger than that of GA. This indicates that the minimum latency can not be obtained using our proposed algorithm. This means that our proposed method cannot obtain the optimal solution for the optimization problem. However, the minimum latency of Proposed is much lower than that of Comp by using appropriate parameters in Algorithm 4. Here, the minimum latency of Random is the lowest among the four methods by ignoring the total latency reduction. In terms of the maximum latency, Figure 7 demonstrates that the latency of Proposed is almost the same as that of GA. This result signifies that Proposed can allocate tasks to appropriate servers so as not to increase the maximum latency for the total latency reduction. Here, the maximum latency for GA, Proposed, and Random is 0.4 that is equal to 2τ, and this is the latency for the task offloading to cloud servers. Therefore, GA, Proposed, and Random can utilizes MEC servers appropriately, but MEC servers are overused in Comp. These results indicate that our proposed heuristic algorithm is effective in solving the optimization problem to reduce the total latency.

**Figure 8:**
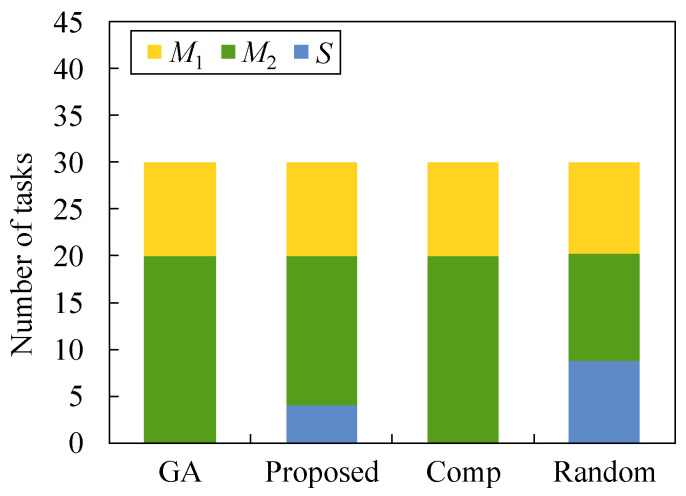
Number of tasks allocated to each server in the case of $N_{1}=30$, τ=0.2, and λ=2.0.

**Figure 9:**
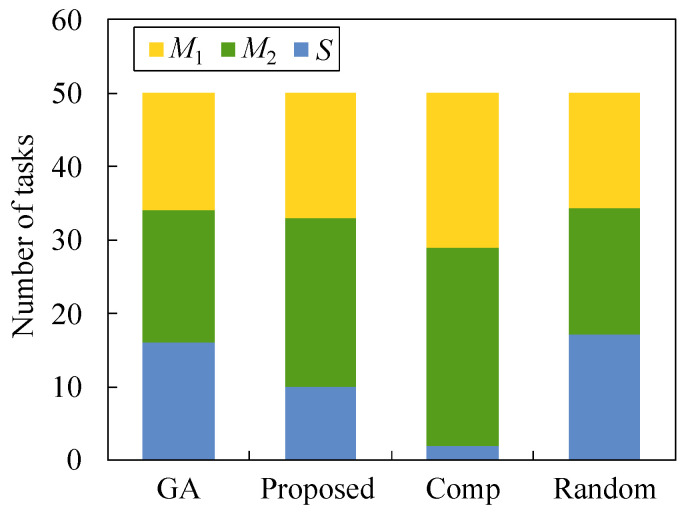
Number of tasks allocated to each server in the case of $N_{1}=50$, τ=0.2, and λ=2.0.

Figure 8 and Figure 9 illustrate how tasks are allocated to each server when *N* is equal to 30 and 50, respectively. In both figures, τ is 0.2 and λ is 2.0. In these figures, almost the same number of tasks are allocated to each server for Random because the task allocation is determined at random. By comparing Random with other methods, we observe that a large number of tasks are allocated to cloud server *S*. As a result, the minimum latency and maximum latency are low in Figure 6 and Figure 7; however, the total latency is high in Figure 5. In our proposed method, the number of tasks for $M_{1}$ is almost the same as that of GA, but the number of tasks for $M_{2}$ and *S* are somewhat different from that for GA. In our proposed method, the number of tasks offloaded to each server depends on the processing order that is predetermined at line 1 in Algorithms 3 and 4. Therefore, it is hard to obtain the optimal task offloading in our proposed method. In Comp, the number of tasks for *S* is the smallest for both cases because the total latency cannot be considered in Algorithm 4.

### 7.2. Impact of Transmission Delay τ for Cloud Server

**Figure 10:**
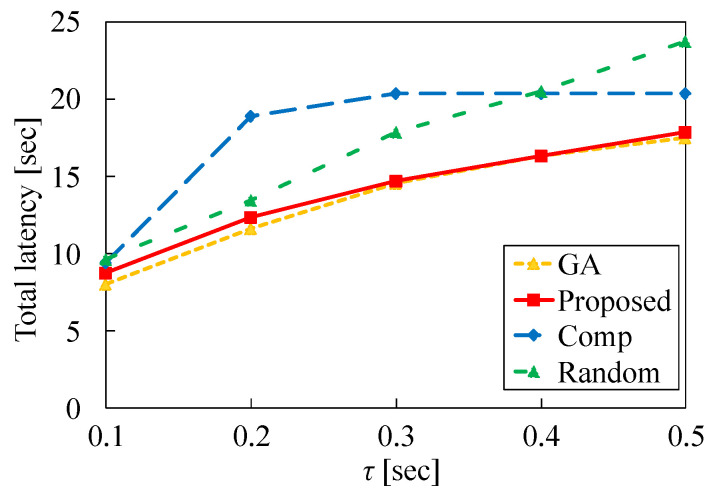
Total latency versus transmission delay τ in the case of $N_{1}=50$ and λ=2.0.

Next, we investigate the impact of the transmission delay τ on the performance of each method. Figure 10 presents the total latency versus τ in the case of $N_{1}=50$ and λ=2.0. This figure indicates that the total latency increases as τ increases for GA, Proposed, and Random. This is because the latency for tasks that are allocated to the cloud server *S* increases. From this figure, we find that the total latency for Proposed is close to that for GA. This means that our heuristic algorithm is effective regardless of τ. In contrast, the total latency of Comp does not increase when τ is larger than 0.2. In Comp, many tasks are allocated to *S* by considering only the latency for each task when τ is small. However, the latency for each task allocated to *S* increases as τ increases. Therefore, the number of tasks for *S* decreases and the total latency does not increase even if τ increases.

**Figure 11:**
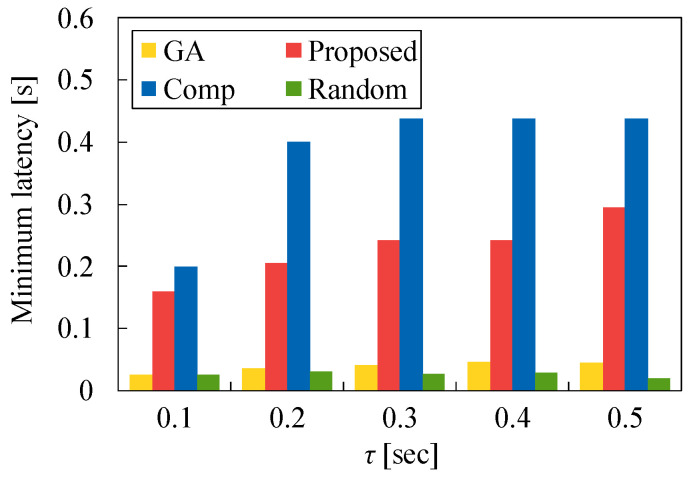
Minimum latency for each method versus τ in the case of $N_{1}=50$ and λ=2.0.

**Figure 12:**
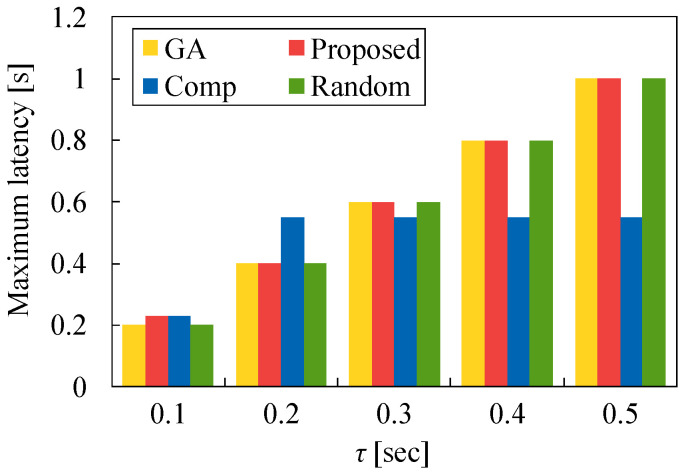
Maximum latency for each method versus τ in the case of $N_{1}=50$ and λ=2.0.

We also evaluate the minimum latency and maximum latency for each task allocation versus τ. In Figure 11 and Figure 12, $N_{1}$ is set to 50 and λ is set to 2.0. Figure 11 demonstrates that the minimum latency of Comp is the largest because many tasks are allocated to *S* even if $M_{1}$ and $M_{2}$ are available. On the other hand, the minimum latency of Proposed is higher than that of GA and Random. The difference between Proposed and these two methods increases as τ increases. This signifies that many tasks are allocated to MEC servers using our proposed method, and the minimum latency of our method increases. However, in Figure 12, the maximum latency of Proposed is equal to that of GA in most cases. In contrast, the maximum latency of Comp is very different from that of other methods. This is because many tasks are allocated to MEC servers even if the processing time in these servers increases. Although the task allocation of Proposed is somewhat different from that of GA, our heuristic algorithm is more effective than Comp and Random.

### 7.3. Impact of Arrival Rate λ in a MEC Platform

In this subsection, we investigate the impact of the arrival rate λ on the performance of each method in the case of $N_{1}=50$ and τ=0.2. The change of arrival rate λ signifies that the change of the number of tasks, and the scalability of our heuristic algorithm and the heterogeneity of system model, can be investigated. It should be noted that there are no results of Random because the obtained random task allocation could not satisfy constraint conditions.

**Figure 13:**
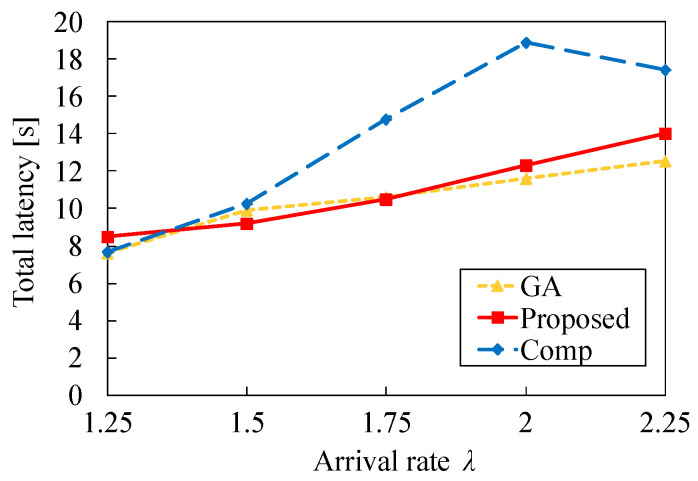
Total latency versus arrival rate λ in the case of $N_{1}=50$ and τ=0.2.

Figure 13 presents the total latency versus λ. This figure indicates that the total latency increases as λ increases for GA and Proposed. This is because the latency $T_{2}^{i}$ increases from (1) and (3). In contrast, when λ increases from 2.0 to 2.25, the total latency of Comp decreases. This is because many tasks are allocated to the cloud server *S* and the processing time on MEC servers decreases. Figure 13 demonstrates that our proposed heuristic algorithm can effectively reduce the total latency compared to Comp regardless of λ.

**Figure 14:**
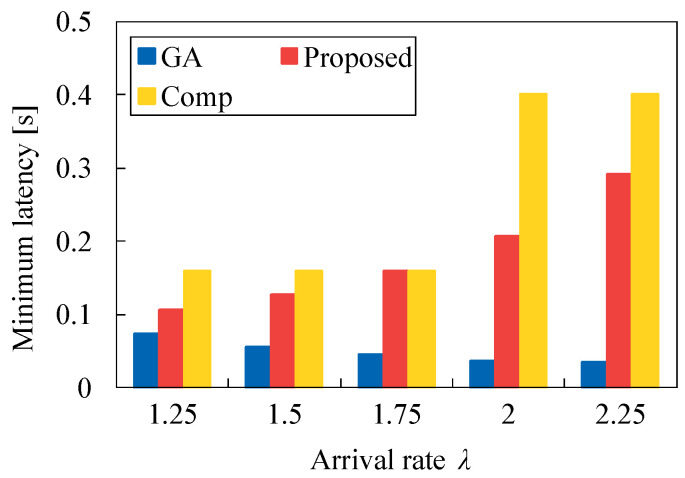
Minimum latency for each method versus λ in the case of $N_{1}=50$ and τ=0.2.

**Figure 15:**
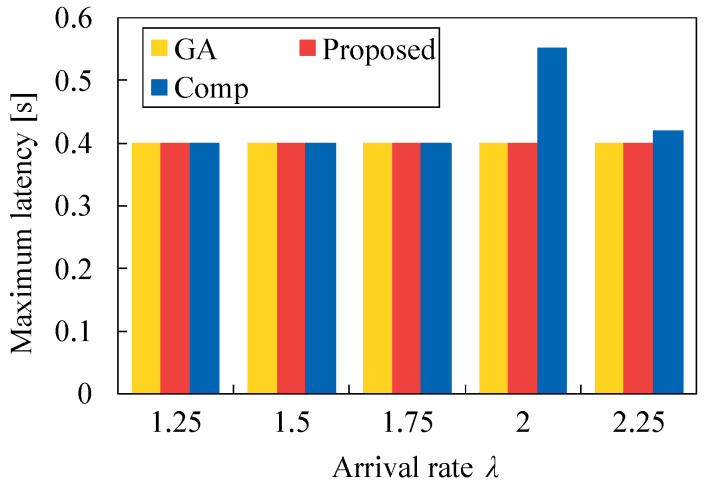
Maximum latency for each method versus λ in the case of $N_{1}=50$ and τ=0.2.

In addition, we evaluate the minimum latency and maximum latency for each task allocation versus λ. Figure 14 demonstrates that the minimum latency of Proposed is higher than that of GA, but is lower than Comp. In Figure 15, the maximum latency of Proposed is equal to that of GA regardless of λ. These results present that our heuristic algorithm is more effective than Comp, although the task allocation of Proposed is somewhat different from that of GA. Here, the proposed method utilizes the total latency, which is given by $K_{i}^{1}$, $K_{i}^{2}$, or $K_{i}^{S}$, in our Algorithm 4, but Comp uses the latency for a server, which is given by $L_{i}^{1}$, $L_{i}^{2}$, or 2τ. This means that our proposed algorithm is effective by considering the total latency instead of the latency for a server. This tendency can be shown in the previous subsection.

### 7.4. Calculation Time

Finally, we investigate the calculation time of our proposed method using a computer running macOS Mojave 10.14.6 with 2.3 GHz Intel Core i5, and 8 GB memory. It should be noted that the calculation time changes every time it is measured and it is not constant.

Table 2 presents the calculation time of GA, Proposed, and Comp in the case of $N_{1}=$ 10, 20, 30, 40, and 50. Here, τ is equal to 0.2 and λ is equal to 2.0. This table indicates that the calculation time of our proposed method (Proposed) is much lower than that of near-optimal task allocation (GA). As the number of tasks increases, the difference between Proposed and GA becomes large. This is because meta-heuristic algorithms including GA take a longer processing time than heuristic algorithms as widely known. This means that the heuristic algorithm is effective for task allocation in real environments. GA is not appropriate to decide the task offloading in real time. Moreover, the calculation time of our proposed method is almost the same as that of another heuristic algorithm (Comp). This is because the two algorithms are almost identical, although our proposed algorithm is more effective. From these results, we can conclude that our proposed heuristic algorithm is effective for task allocation in a MEC platform with multiple types of MEC servers.

## 8. Conclusions and Future Work

For 5G and future Internet, in this paper, we proposed a task allocation method for reducing the total latency in a MEC platform with three types of servers: a dedicated MEC server, a shared MEC server, and a cloud server. The proposed method can perform approximate optimal task allocation in a shorter time than other meta heuristic algorithms. This heuristic algorithm consists of four algorithms: a main algorithm and three additional algorithms. In this algorithm, tasks are divided into two groups, and task allocation is executed for each group. Computational complexity of our proposed algorithm depends on only the number of tasks. We compared the performance of our proposed heuristic algorithm with the solution obtained by GA and evaluated the effectiveness of our algorithm. From numerical examples, we observed that the results of our proposed method were similar to the results of near-optimal task allocation with GA. When the number of tasks changed, the difference between our proposed method and GA did not change significantly. In addition, the proposed algorithm could reduce the total latency by comparing with other methods. In terms of the transmission delay, the effectiveness of the proposed method was much high even if the transmission delay increased. This is because our proposed method can utilize MEC servers as is the case with GA. On the other hand, as the arrival rate became large, the difference between the proposed method and GA increased. This is because the impact of incorrect task allocation became large as the arrival rate increased. Nevertheless, the proposed method was more effective than other methods. The calculation time of our proposed method was much lower than that of near-optimal task allocation with GA. This result signified that Proposed could allocate tasks to appropriate servers so as not to increase the maximum latency for the total latency reduction. These results indicated that our proposed heuristic algorithm was effective in solving the optimization problem to reduce the total latency. Our proposed heuristic algorithm was effective for task allocation in a MEC platform with multiple types of MEC servers.

For a large-scale MEC platform, our system model and proposed algorithm are utilized modeling multiple MEC servers and multiple access points as a shared MEC server and an access point group, respectively. If the impact of each MEC server and each access point should be evaluated, our proposed method must be extended. This extension is one of our future works. In addition, we have developed an open MEC platform and a mobile augmented reality application. In our future work, we will implement the proposed algorithm into our MEC platform and mobile application and experimentally evaluate the performance of the algorithm. Moreover, in order to improve the performance, a deep learning algorithm may be available in the future.

## Figures and Tables

**Table 1:** Parameter settings in simulation.

Parameter	Value
Number of tasks transmitted via access point $a_{1}$	$N_{1}=10$, 20, 30, 40, or 50
Number of tasks transmitted via access point $a_{2}$	$N_{2}=20$
Processing efficiency for MEC server $M_{1}$	$D_{1}=30$
Processing efficiency for MEC server $M_{2}$	$D_{2}=300$
Transmission delay of tasks for *S*	τ=0.1, 0.2, 0.3, 0.4, or 0.5
Task size $c_{i}$	Uniform distribution of [0.1,1.0]
Acceptable latency $t_{i}^{max}$	Uniform distribution of [1.0,4.0]
Arrival rate λ of tasks for M/M/1 queueing model	λ=1.25, 1.5, 1.75, 2.0, or 2.25
Average processing time of task for M/M/1 queueing model	$\frac{1}{\mu }=0.01$

**Table 2:** Calculation time of each method in the case of τ=0.2 and λ=2.0.

Number of Tasks $N_{1}$	GA [s]	Proposed [s]	Comp [s]
10	1004.345	0.093	0.092
20	1048.478	0.184	0.126
30	1720.223	0.266	0.246
40	3995.914	0.342	0.319
50	4983.124	0.411	0.316

## Data Availability

Not applicable.
